# In Silico Screening of *Cannabis sativa* Phytochemicals as Potential Ornithine Decarboxylase Inhibitors for Anti-Leishmanial Drug-Prioritized Compound Development

**DOI:** 10.3390/biology15151272

**Published:** 2026-08-03

**Authors:** Abdul Haseeb Khan, Munazza Kanwal, Syed Babar Jamal, Kashaf Maheen, Sunia Mohsin, Laiba Hussain, Fawaz Al-Hussain, Kaleem Imdad, Shumaila Naz, Shahid Bashir

**Affiliations:** 1 Department of Biotechnology, National University of Medical Sciences, Rawalpindi 46000, Pakistan; abdulhaseebkhan6666@gmail.com (A.H.K.); munazzakanwal83@gmail.com (M.K.); kashafmaheen03@gmail.com (K.M.); suniamohsin24@gmail.com (S.M.); hlaiba812@gmail.com (L.H.); 2 Department of Molecular Biology & Genetics, National University of Medical Sciences, Rawalpindi 46000, Pakistan; babar.jamal@numspak.edu.pk; 3 Department of Neurology & Stroke, College of Medicine, King Saud University, Riyadh 11421, Saudi Arabia; faalhussain@ksu.edu.sa; 4 Department of Biosciences, COMSATS University Islamabad, Islamabad 45550, Pakistan; 5 Neuroscience Center, King Fahad Specialist Hospital, Dammam 32253, Saudi Arabia; sbashir10@gmail.com

**Keywords:** *Cannabis sativa*, phytochemicals, ornithine decarboxylase, *Leishmania*, molecular docking, molecular dynamics simulations, anti-leishmanial prioritized compound discovery

## Abstract

Leishmaniasis remains a major neglected tropical disease with limited therapeutic options, increasing drug resistance, and significant treatment-associated toxicity. Ornithine decarboxylase (ODC), which plays an essential role in polyamine synthesis and survival of parasites, is a potential molecular target for the discovery of anti-leishmanial agents. In this study, 49 natural products with bioactive properties, such as cannabinoids, terpenoids, flavonoids, polyphenols, and alkaloids, are evaluated against ODC using computational approaches like molecular docking and molecular dynamics (MD) simulations. During molecular docking analysis, some compounds showed good affinity binding to the ODC catalytic site, namely Sanguinarine (−8.53 kcal/mol), Rutin (−8.15 kcal/mol), Evodiamine (−7.83 kcal/mol), Cannabinol (−7.58 kcal/mol), and β-sitosterol (−7.56 kcal/mol). Analysis of protein-ligand complex interactions showed that these compounds formed hydrogen bonds, hydrophobic interactions, π-alkyl contacts, π-cation contacts, and van der Waals forces in the vicinity of the active-site amino acid residue. For further analysis, MD simulations were performed for 100 ns on the best-docking complexes. Comparative trajectory analysis of RMSD, RMSF, Rg, SASA, and hydrogen bonds was conducted, revealing that Rutin and Evodiamine exhibited relatively high structural stability and consistent interactions within the ODC binding cavity. Overall, this study indicates that the natural compounds analyzed here could be considered promising hit compounds for anti-leishmanial drug discovery against ODC. However, further investigations using experimental techniques are required to confirm their biological properties and efficacy.

## Simple Summary

Leishmaniasis is considered a neglected tropical disease due to limited treatment options, increasing resistance to prioritized compounds, and high toxicity associated with treatment, necessitating the exploration of new potential treatment approaches with improved safety profiles. In this research, computational tools were used to evaluate the efficacy of certain natural bioactive compounds on Ornithine Decarboxylase, which is a key enzyme involved in parasite viability. Molecular docking found several compounds, including Sanguinarine, Rutin, Evodiamine, Cannabinol, and β-sitosterol, with good binding energy and interaction patterns. Further, molecular dynamics studies showed that Rutin and Evodiamine had relatively stable interactions with the protein target. Overall, the current findings suggest that selected natural compounds could serve as potential candidates for prioritized compound development in the treatment of leishmaniasis.

## 1. Introduction

Leishmaniasis is a neglected tropical disease caused by protozoa of the genus *Leishmania*, transmitted through the bites of infected female sandflies of the genus Phlebotomus [1,2]. The disease affects populations in more than 90 countries, with an estimated 0.7–1 million new cases reported annually, particularly in tropical and subtropical regions [3]. Clinically, leishmaniasis manifests in three major forms: cutaneous, mucocutaneous, and visceral leishmaniasis [4]. Among these, cutaneous leishmaniasis (CL) is the most prevalent and is characterized by ulcerative skin lesions that may lead to permanent scarring [5], whereas visceral leishmaniasis is the most severe form and can be fatal if left untreated [6].

Despite decades of research, the control and treatment of leishmaniasis remain challenging due to multiple factors, including limited access to healthcare, socioeconomic constraints, and the emergence of drug-resistant *Leishmania* strains [7]. Current therapeutic options, such as pentavalent antimonials, amphotericin B, miltefosine, and paromomycin, are associated with high toxicity, prolonged treatment regimens, and increasing resistance [8,9]. Although difluoromethylornithine (DFMO) has been successfully used as an inhibitor of ornithine decarboxylase (ODC) in African trypanosomiasis, its application against *Leishmania* is limited, highlighting the need for alternative ODC-targeting compounds with improved efficacy and safety profiles [10].

Ornithine decarboxylase (ODC) is a rate-limiting enzyme in the polyamine biosynthesis pathway, catalyzing the decarboxylation of ornithine to putrescine, a precursor for spermidine and spermine. These polyamines are essential for parasite growth, nucleic acid stabilization, and resistance to oxidative stress [11,12]. ODC inhibition disrupts polyamine homeostasis, thereby impairing parasite survival and proliferation. Given its essential role and draggability, ODC represents a well-validated molecular target for the development of anti-leishmanial therapies [13,14].

Advancements in computational biology have accelerated early-stage drug discovery by enabling rapid screening of large compound libraries through structure-based approaches [15]. Molecular docking and molecular dynamics simulations are widely used to predict ligand-protein interactions, binding affinities, and conformational stability. These in silico techniques provide a cost-effective platform for identifying potential hit compounds prior to experimental validation [16,17].

Natural products continue to play a vital role in drug compound discovery due to their structural diversity and broad spectrum of biological activities. *Cannabis sativa* (*C. sativa*) is a medicinal plant known to contain a rich repertoire of bioactive phytochemicals, including cannabinoids, terpenoids, flavonoids, and polyphenols [18,19]. Several of these compounds have demonstrated antimicrobial, antiparasitic, anti-inflammatory, and immunomodulatory properties [20,21]. Emerging evidence suggests that cannabinoids and related phytochemicals may interfere with protozoan parasites by disrupting key metabolic pathways, modulating oxidative stress, and altering host–parasite interactions [22,23]. However, their potential role in targeting ODC in *Leishmania* remains largely unexplored.

In this context, the present study aims to investigate selected phytochemicals derived from *C. sativa* as potential ODC inhibitors using an integrated in silico approach [24,25]. A total of 49 compounds, representing diverse chemical classes, were screened using molecular docking to evaluate their binding affinity for the ODC active site. The most promising candidates were further analyzed through molecular dynamics simulations to assess the stability of protein-ligand interactions over time. While computational predictions provide valuable preliminary insight, the findings of this study are intended to serve as a foundation for future experimental validation through in vitro and in vivo studies.

## 2. Materials and Methods

**Figure 1:**
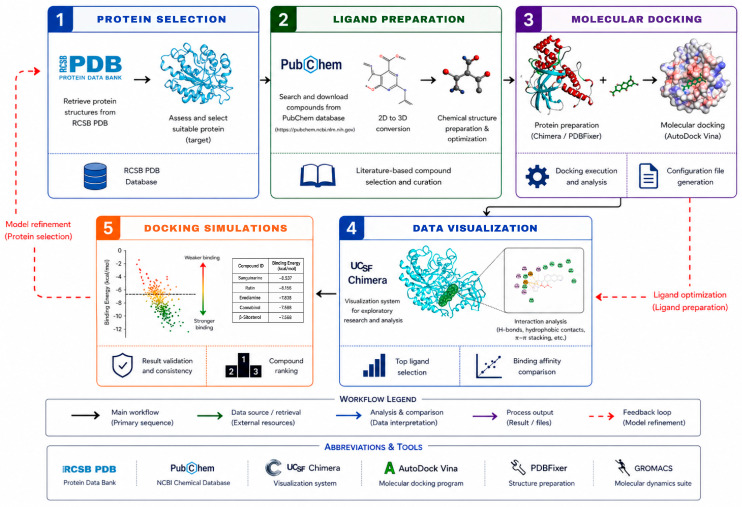
Molecular docking workflow: select and prepare protein, prepare ligands, dock, analyze interactions/scores, visualize results.

The design of a Molecular docking workflow involved numerous technique steps. Figure 1 provides a summary of the general process and pipeline used in the current study.

### 2.1. Protein Structure Modeling

The three-dimensional structure of Ornithine Decarboxylase (PDB ID: 9FSC) was retrieved directly from the RCSB Protein Data Bank [26] (https://www.rcsb.org/) (accessed on 3 February 2026). The crystal structure was obtained in PDB format and subsequently prepared for molecular docking. During receptor preparation, water molecules and co-crystallized ligands were removed to isolate the protein structure using Discovery Studio 2024 (Dassault Systèmes BIOVIA, San Diego, CA, USA). Polar hydrogen atoms were added to the receptor, and Gasteiger partial charges were assigned using OpenBabel v3.1.1 (Open Babel development team, open-source; https://openbabel.org) (accessed on 3 February 2026). Non-polar hydrogen atoms were removed to reduce steric artifacts during docking, retaining only polar hydrogens essential for hydrogen bond calculations. The prepared receptor was saved in PDBQT format for compatibility with AutoDock Vina v1.2.5 (The Scripps Research Institute, La Jolla, CA, USA).

### 2.2. Structural Validation of Protein

To ensure the structural integrity of the selected model, the following validation tools were used:

- ERRAT Score Calculation: The ERRAT tool available on the UCLA SAVES server (UCLA-DOE Institute, University of California, Los Angeles, CA, USA; https://saves.mbi.ucla.edu/) (accessed on 3 February 2026) was employed to identify regions of erroneous non-bonded atomic interactions [27]. A high ERRAT score reflects good model reliability.
- Ramachandran Plot Analysis: MolProbity (Richardson Laboratory, Duke University, Durham, NC, USA; http://molprobity.biochem.duke.edu/) (accessed on 3 February 2026) was used to perform Ramachandran analysis [28]. This analysis compared the backbone dihedral angles of amino acid residues, which assisted in evaluating the stereochemical quality of the model [29].
- Z-score and Local Model Quality Estimation: The ProSA-web tool (Center of Applied Molecular Engineering, University of Salzburg, Salzburg, Austria; https://prosa.services.came.sbg.ac.at/prosa.php) (accessed on 3 February 2026) was utilized to compute the Z-score of the model, and the model was evaluated against the structures of related size in a protein structure database to measure their quality [27]. A Z-score check across the native protein set indicates the validity of the anticipated model [30]. Residue-wise energy plots were also provided by the ProSA tool to identify local regions of instability or poor-quality predictions within the model.

### 2.3. Preparation of Ligands for Molecular Docking

49 bioactive products were identified in this analysis and subjected to molecular docking against ornithine decarboxylase (ODC), based on literature reports of antiproliferative/anticancer activity, ethnopharmacological relevance, and chemical diversity (Tables S1–S6, Supplementary Material). These compounds were classified into five broad categories: cannabinoids (19), terpenoids (10), flavonoids (8), polyphenols (5), and alkaloids (7). The compounds were obtained in SDF format from PubChem (National Center for Biotechnology Information, National Library of Medicine, Bethesda, MD, USA; https://pubchem.ncbi.nlm.nih.gov/) (accessed on 3 February 2026) along with their respective PubChem CIDs: Sanguinarine (PubChem CID: 5154), Rutin (PubChem CID: 5280805), Evodiamine (PubChem CID: 442088), Cannabinol (PubChem CID: 2543), and β-Sitosterol (PubChem CID: 222284). The ligand structures were energy-minimized using the MMFF94 (Merck Molecular Force Field 94; Merck & Co., Inc., Kenilworth, NJ, USA) force field implemented in RDKit 2024.03 (RDKit community, open-source cheminformatics; https://www.rdkit.org) (accessed on 3 February 2026), and protonation states were corrected at physiological pH 7.4 using Dimorphite-DL (Durrant Lab, University of Pittsburgh, Pittsburgh, PA, USA). The structures were subsequently converted to PDBQT format using OpenBabel v3.1.1 for compatibility with AutoDock Vina [31].

### 2.4. Molecular Docking

Subsequently, molecular docking was performed using AutoDock Vina v1.2.5 (The Scripps Research Institute, La Jolla, CA, USA) to assess ligand orientation, binding affinity, and interactions with the active site of ornithine decarboxylase [32]. The druggable binding pocket was identified using fpocket v3 (Institute Pasteur, Paris, France), in which the most druggable pocket (Pocket #1) was confirmed by a druggability score of 0.987, and the key residues identified (LYS288, CYS385, PHE387, ARG375, HIS418, TYR663) correspond to the known ODC catalytic site. The grid box was centered at (−12.990, −46.182, −9.320), with dimensions of 28 × 26 × 30, and an exhaustiveness of 16. Nine binding modes were predicted for each ligand. The protein (receptor) was prepared in PDBQT format with polar hydrogens retained. Both the ligand and the protein were obtained in PDBQT file formats for docking.

### 2.5. Visualization and Analysis

The ligand/receptor docked complex was subsequently analyzed and visualized using Discovery Studio 2024 (Dassault Systèmes BIOVIA, San Diego, CA, USA), UCSF Chimera (Resource for Biocomputing, Visualization, and Informatics, University of California, San Francisco, CA, USA), and Icn3D (National Center for Biotechnology Information, National Library of Medicine, Bethesda, MD, USA) [32,33,34]. Label functions were applied to explain the residues, atoms, and area of interest. In this step, emphasis was placed on amino acids involved in receptor binding. Next, hydrogen bonds and noncovalent interactions, such as hydrophobic, electrostatic, and Van der Waals forces, between the ligands and receptors were analyzed. A 2D interaction map was also generated to summarize the binding interactions. Finally, the docking scores from AutoDock Vina were evaluated, and the binding affinities of all 49 ligands were compared to identify the most promising candidates. According to the binding affinities obtained, Sanguinarine (−8.537 kcal/mol), Rutin (−8.156 kcal/mol), Evodiamine (−7.838 kcal/mol), Cannabinol (−7.588 kcal/mol), and β-Sitosterol (−7.568 kcal/mol) were shortlisted as the top five most promising candidates against ODC.

### 2.6. Docking Interpretation

To confirm that the process had gone down the correct path, the docked ligand complex of the native structure was shown. Root Mean Square Deviation (RMSD) values reported in the docking results are relative to the best docking pose (Mode 1, the pose with the lowest binding energy) as generated by AutoDock Vina v1.2.5. Mode 1 is assigned an RMSD of 0.000 Å by definition, and all subsequent modes (2–9) are reported as RMSDs from this reference pose [35]. A low RMSD between modes indicated minor conformational deviation, confirming pose consistency during docking [36]. This was followed by further analysis and comparison of compounds with high binding affinity for ODC to clarify their interaction patterns and binding residues [37].

### 2.7. Molecular Dynamics Simulation Analysis

All MD simulations were run with GROMACS 2023 (GROMACS development team, KTH Royal Institute of Technology, Stockholm, Sweden; https://www.gromacs.org) (accessed on 3 February 2026). The protein structure of ornithine decarboxylase was analyzed using the pdb2gmx module force field. The ligands were parameterized with ACPYPE (open-source; https://github.com/alanwilter/acpype) (accessed on 3 February 2026) using GAFF/AM1-BCC charges, and the resulting ligand topology was used in GROMACS. The compound was enclosed in a dodecahedral box and solvated with TIP3P water molecules. Using the genion module, we added Na^+^/Cl^−^ ions to neutralize and set the ionic strength to 0.15 M. The steepest-descent algorithm was used to minimize energy until a maximum force of less than 10 kJ/mol/nm was reached.

Equilibration included a 100 ps NVT phase utilizing the V-rescale thermostat at 300 K, followed by a 100 ps NPT phase using the Parrinello-Rahman barostat at 1 bar. Throughout equilibration, all heavy atoms remained restrained in their positions. Periodic boundary conditions were imposed in both directions, long-range electrostatics were calculated using Particle Mesh Ewald (PME), and all hydrogen-containing covalent bonds were restricted using the LINCS algorithm.

A 100 ns production MD simulation was run with a 2-fs time step, and coordinates were saved every 10 ps. Trajectory analyses were conducted using standard built-in GROMACS tools. Trajectory graph analyses were plotted using XMGrace version 5.1.25 (open-source; https://plasma-gate.weizmann.ac.il/Grace) (accessed on 3 February 2026). Three-dimensional trajectory visualization and protein–ligand interaction analysis were performed using UCSF Chimera and Discovery Studio 2024 (Dassault Systèmes BIOVIA, San Diego, CA, USA).

## 3. Results

### 3.1. Protein Modeling and Structural Validation

**Figure 2:**
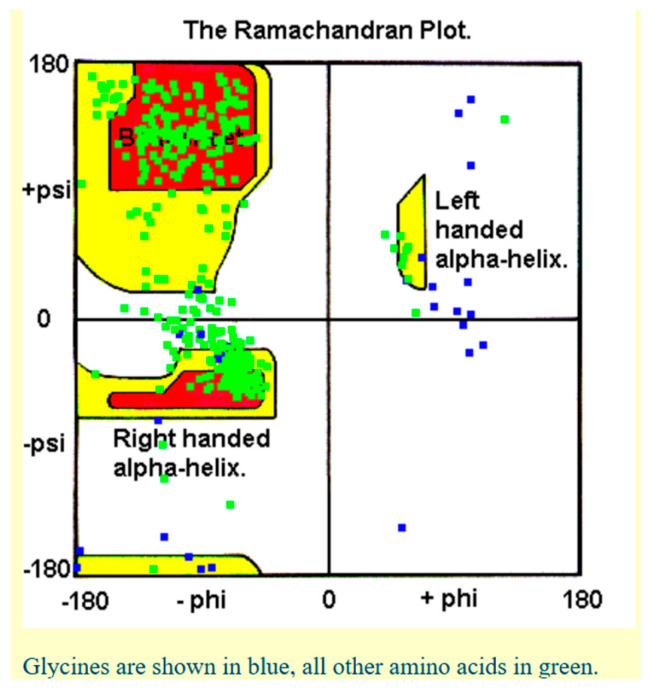
Ramachandran plot of the ODC receptor model, as provided by MolProbity, showing the distribution of residues across favored, allowed, and outlier regions.

The ODC three-dimensional structure (PDB ID: 9FSC) was retrieved from PDB [38]. Extensive validation was conducted to ensure the model’s validity and precision. The Ramachandran plot showed that 92–95% of residues fall within an acceptable range, indicating excellent stereochemical integrity. Also, the ERRAT analysis produced a quality factor of 96.02, depicting a high-quality model. The ProSA Z-score of −10.92 was within the range of native proteins, and local model quality plots also indicated structural stability. All these validation findings support the claim that the constructed ODC structure is sound and can be used as a receptor in molecular docking. These results suggest that the model is of high-quality [28]. A well-folded model and trustworthy backbone torsion angles are affirmed by a clear region occupancy [39]. The presence of a few glycine and serine residues in loop regions, along with those in outlier regions, is permissible due to their flexibility [40]. This finding supports the integrity of the docking receptor shown in Figure 2.

#### 3.1.1. ERRAT2 Quality Factor

**Figure 3:**
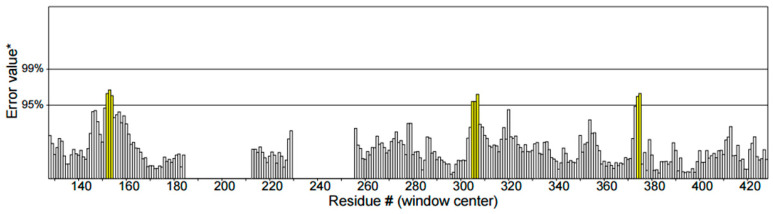
Error plot ERRAT2 of the residues 1–300 of the ODC receptor with areas of large and small errors. (Residue # means residue number or amino-acid position in the protein sequence. Here # simply means “number.” The * beside ERRAT Value is a footnote/reference marker used by the ERRAT output. It does not indicate statistical significance).

**Figure 4:**
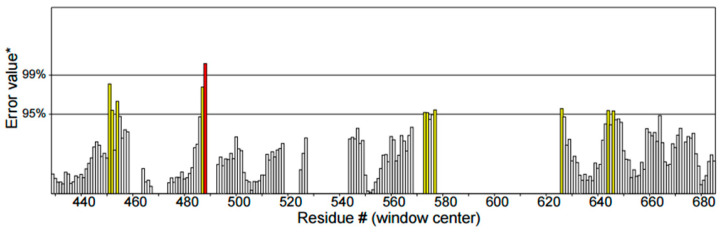
ERRAT 2 plot of residues 300 to 450 of the ODC receptor, showing a high error with localized error peaks, but a high model quality overall. (Residue # means residue number or amino-acid position in the protein sequence. Here # simply means “number.” The * beside ERRAT Value is a footnote/reference marker used by the ERRAT output. It does not indicate statistical significance).

After ERRAT2 analysis, the quality score was 95.411%, exceeding the commonly used standard of 90% for excellent crystallographic models [27,41]. The plotted error values in Figure 3 and Figure 4 show a very low proportion of values exceeding the 95% confidence threshold, and the structural stability of the protein core is only slightly affected by changes in the loop regions. The result verifies that the model is of high-quality and can be used for docking analysis.

#### 3.1.2. ProSA-Web Z-Score Analysis

**Figure 5:**
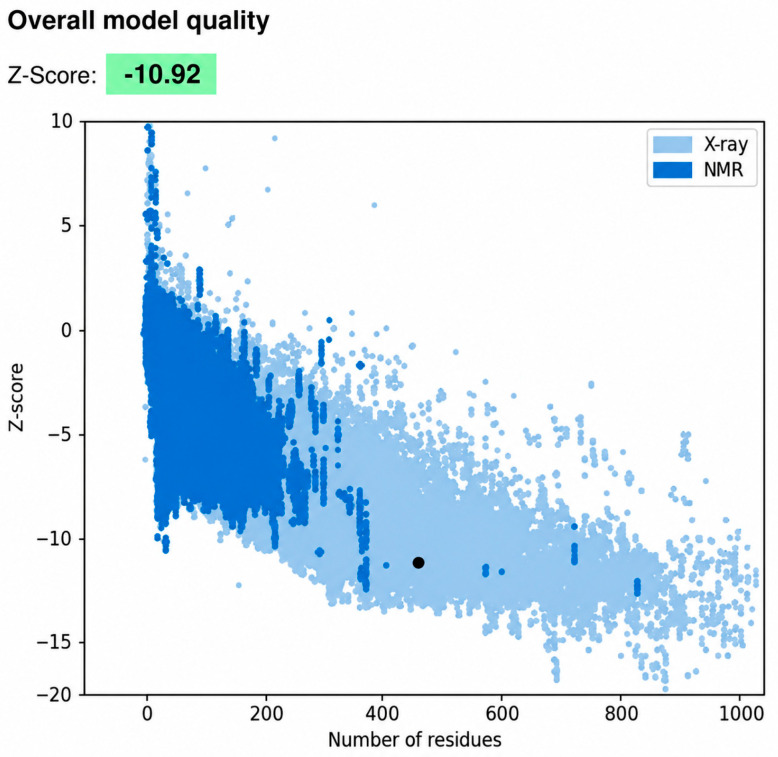
ProSA-web Z-score plot of the ODC receptor, the score in comparison to the experimentally known protein structures in the Protein Data Bank (PDB). The black dot indicates the region where the protein structure lies, either in X-ray or NMR.

The ProSA analysis yielded a Z-score of −10.92, which falls within the range typically observed for experimentally determined protein structures, particularly those analyzed by X-ray crystallography or NMR spectroscopy [42]. The ProSA Z-score plot (Figure 5) shows that the protein model is well aligned with the distribution of known structures in the Protein Data Bank (PDB), implying that the model’s general fold and energy structure are similar to those of native proteins [26]. Moreover, findings from local alignment-independent analysis indicate that the structure fits well within the cloud of validated PDB entries, implying that it lacks large-scale structural aberrations. Taken together, these results showed that the modeled protein has a good energy profile and structural stability fit, and it can therefore be subjected to downstream computational studies.

#### 3.1.3. Local Model Quality

**Figure 6:**
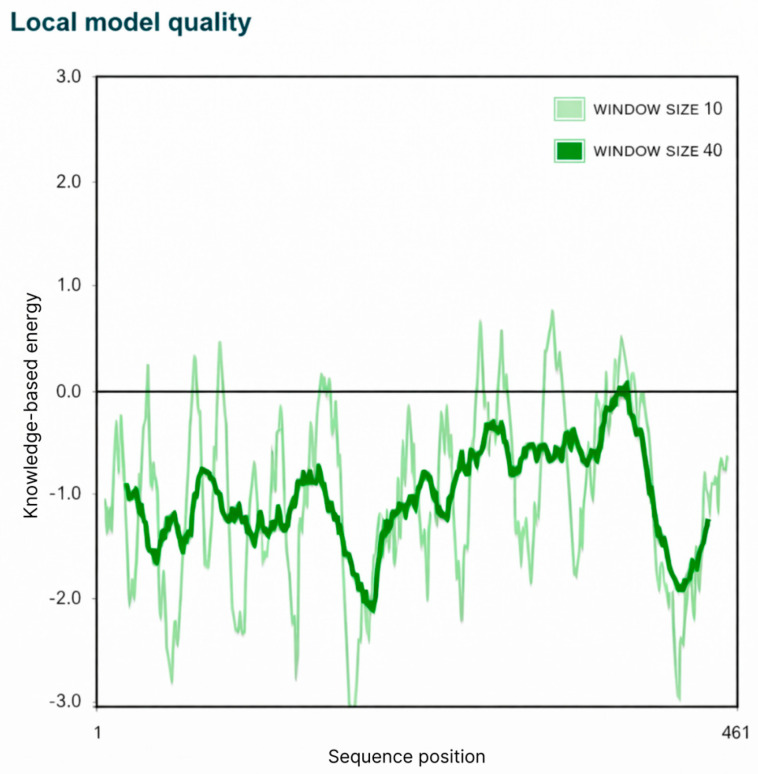
It demonstrates knowledge-based energy using sequence positions and measures it for two local regions (10 positions and 40 positions).

The local model quality, as illustrated in Figure 6, gives the knowledge-based energy profile across the sequence positions of the protein. There are also slight changes to the plot of green energy, but these changes do not exceed the usual energy limits, meaning that the structure is generally stable [43]. The WINDOW SIZE 40 dark green curve is a smoother depiction of the distribution of the energy, and therefore, the areas with comparatively more or less energy states can be identified better [42]. The negative values of the energy dominate the length of the sequence represent the stable and compact protein conformation. In general, this analysis supports the validity of the modeled structure, which shows that all residues occur in energetically favorable conditions.

#### 3.1.4. Icn3D Representation

**Figure 7:**
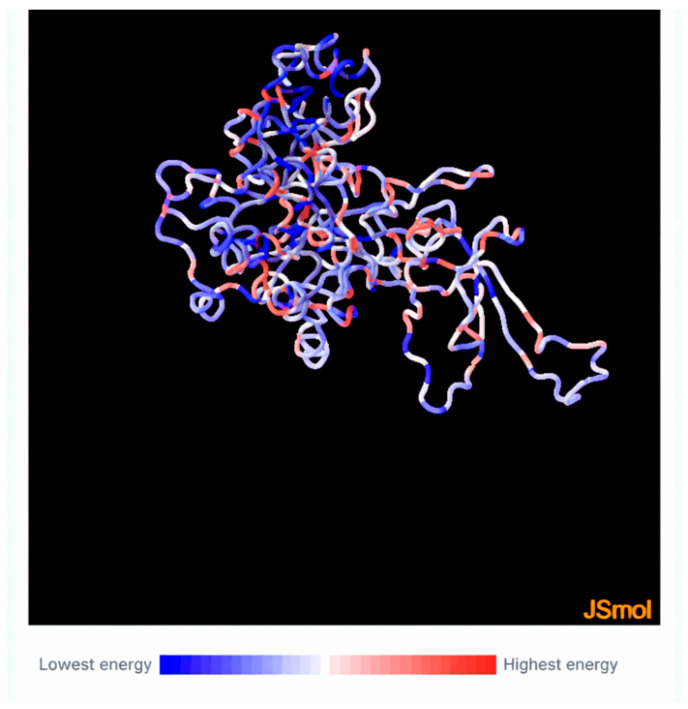
The coloring of the 3D model ranges from blue (the lowest energy level) to red (the highest energy level) on the protein.

The overall structural stability and energy distribution of the protein were visualized using Icn3D. Figure 7 shows the protein’s three-dimensional structure, displayed in different colors based on its energy distribution. The color gradient that moves from blue to red depicts the low and high energy conditions, respectively. The presence of blue and white patches in the middle of the protein indicates the presence of low-energy conformations, a manifestation of high structural stability. By contrast, the regions of red coloration are mostly localized in the loop segments and indicate elevated energy and increased conformational flexibility. This result further validates the findings by the complementary tools used in the study previously.

### 3.2. Ligand Preparation

The present investigation evaluated 49 bioactive compounds, categorized into five major classes: cannabinoids (19), terpenoids (10), flavonoids (8), polyphenols (5), and alkaloids (7). All selected compounds were retrieved from the PubChem database along with their PubChem CIDs, and their structures were prepared through energy minimization and protonation correction prior to docking. Phytochemicals from *C. sativa* have been reported to have various pharmacological effects, including antiparasitic, anti-inflammatory, and antioxidant activities [44]. The binding affinities of these compounds for ornithine decarboxylase were determined to identify the most promising candidates for developing new anti-leishmanial prioritized compounds. The best compounds of each of the four bioactive classes are summed up in Table 1.

### 3.3. Molecular Docking and Interaction Analysis

Molecular docking was performed for 49 phytochemicals from *Cannabis sativa* against ornithine decarboxylase (ODC; PDB ID: 9FSC) using AutoDock Vina software. The druggable binding pocket was identified using fpocket v3 software, in which the most druggable pocket (Pocket #1) was identified by a druggability score of 0.987. In the molecular docking study, the grid box was centered at (−12.990, −46.182, −9.320), with dimensions of 28 × 26 × 30, and an exhaustiveness of 16. Nine binding modes were predicted for each ligand, and the mode having the lowest docking score (Mode 1) was chosen for further evaluation.

Binding threshold values were determined to categorize molecules as strong, moderate, or weak binders. Interestingly, 20 of 49 molecules (approximately 40.8%) showed strong binding affinity, highlighting the potential of natural products as ODC inhibitors.

**Figure 8:**
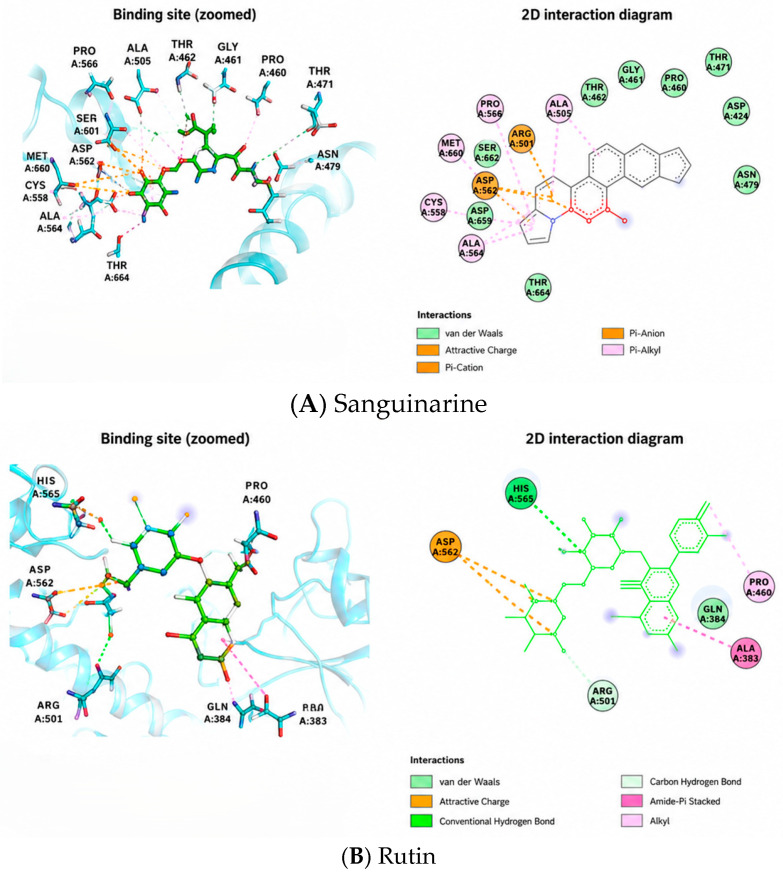
Binding conformations and their corresponding interaction diagrams of the two top-ranking ligands found inside the binding pocket of ornithine decarboxylase (ODC; PDB ID: 9FSC) are shown below. The top image is for (**A**) Sanguinarine, while the bottom image is for (**B**) Rutin. Both molecules occupy the binding pocket identified by the Fpocket server, and they contact key residues in the protein’s active site. Sanguinarine of π-cation, π-alkyl, and van der Waals interactions. In contrast, Rutin exhibits hydrogen bonding and electrostatic interactions.

**Figure 9:**
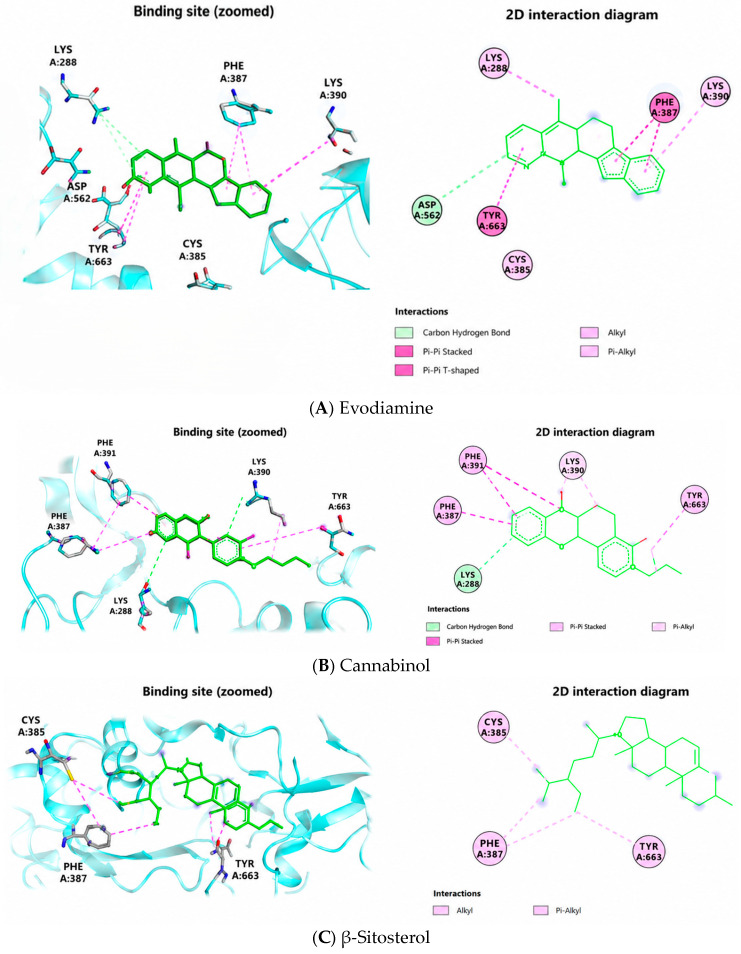
Three-dimensional binding poses and corresponding two-dimensional interaction maps of Evodiamine (**A**), Cannabinol (**B**), and β-sitosterol (**C**) within the ODC active site (PDB ID: 9FSC). All ligands occupy the predicted binding pocket and are primarily stabilized by hydrophobic and π-based interactions, with Evodiamine additionally exhibiting π–π stacking interactions.

For clinical reference, difluoromethylornithine (DFMO), the established ODC inhibitor used in African trypanosomiasis, was included as a benchmark. DFMO showed a binding affinity of −5.8 kcal/mol against ODC, which is notably weaker than all five top-ranked natural compounds identified in this study. In the above-listed compounds, Sanguinarine has the highest binding affinity of −8.53 kcal/mol, followed by Rutin (−8.15 kcal/mol), Evodiamine (−7.83 kcal/mol), Cannabinol (−7.58 kcal/mol), and β-sitosterol (−7.56 kcal/mol) as indicated in Figure 8 and Figure 9. These five molecules were subjected to further studies, viz., MD simulations, to examine their dynamic behavior within the active site pocket.

It is found that the higher binding affinity of those compounds is due to favorable stabilization interactions between the ligands and the target receptor protein. For instance, two ligands, Sanguinarine and Rutin, containing functional groups such as hydroxyl, carbonyl, and amino groups, have higher binding affinities owing to interactions such as hydrogen bonding and π-stacking. On the contrary, β-sitosterol, containing a single functional group, interacts mainly via hydrophobic interaction only.

The data is summarized in Table 1. Based on the aforementioned information, it can be inferred that a few natural products bind to ODC with significant affinity, and their binding behavior requires further confirmation through MD simulations.

### 3.4. Molecular Dynamics Simulation

To analyze the molecular dynamics stability and interactions ODC protein in complex with five small-molecule ligands: Sanguinarine, Rutin, Evodiamine, Cannabinol, and β-sitosterol. Various trajectory analyses, including RMSD, RMSF, Rg, SASA, and hydrogen-bond interactions, were performed to assess structural stability and ligand effects.

**Figure 10:**
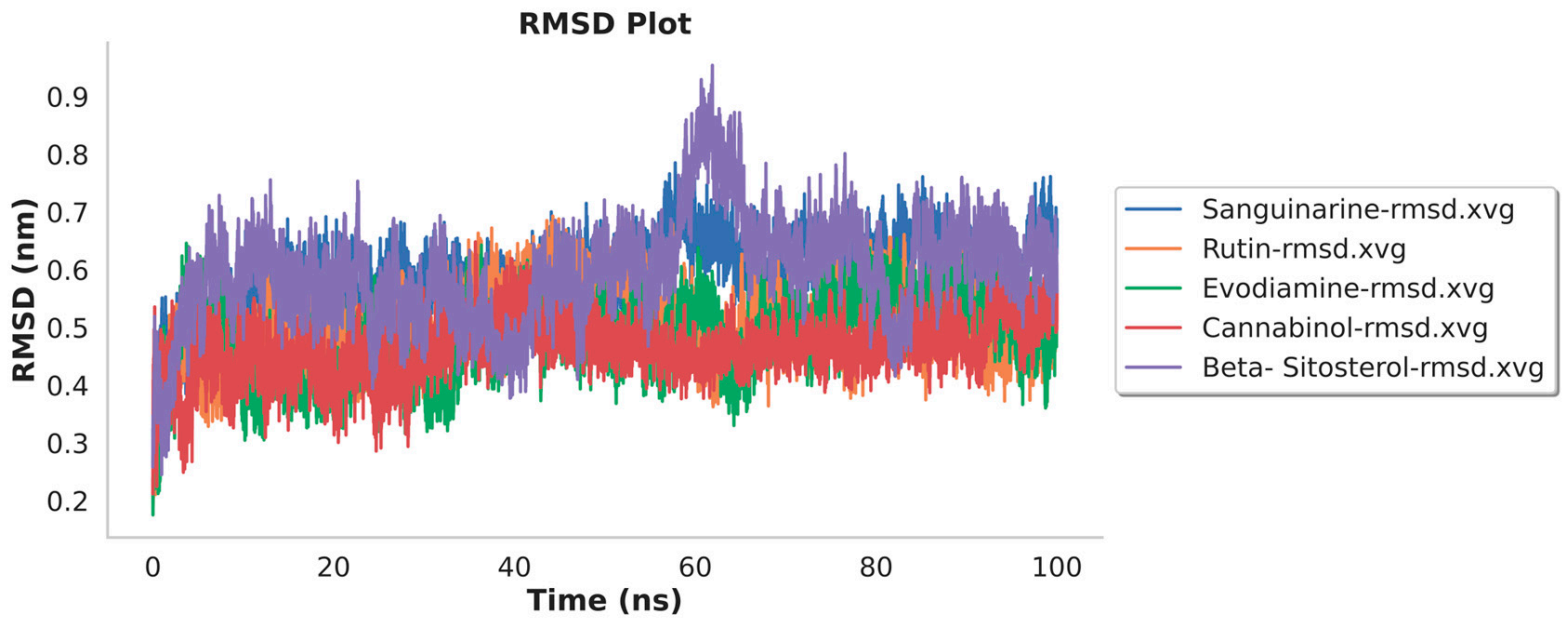
Protein backbone RMSD analysis of ODC–ligand complexes. The backbone RMSD analysis for ornithine decarboxylase (ODC) in complex with Sanguinarine, Rutin, Evodiamine, Cannabinol, and β-sitosterol over a 100 ns molecular dynamics simulation. All systems reached equilibrium after the initial phase, with Evodiamine and Cannabinol exhibiting lower RMSD fluctuations, indicating greater structural stability, while β-sitosterol showed comparatively higher deviation, suggesting increased conformational flexibility.

The RMSD was calculated for all the systems to study their structural stability. It was observed that all the systems initially showed an upward trend, followed by fluctuations at lower levels, indicating the formation of equilibrium among them, as indicated in Figure 10. Of the five complexes studied, the two complexes, Evodiamine and Cannabinol, showed the least RMSD fluctuations, mainly ranging from 0.40 to 0.55 nm, indicating their increased stabilization with the ODC backbone. Meanwhile, the two complexes bound to Rutin and Sanguinarine showed moderate RMSD fluctuations, ranging from 0.45 to 0.65 nm, suggesting acceptable structural stability. On the other hand, the complex bound with β-sitosterol had higher RMSD levels, especially around 60 ns (~0.90 nm). In summary, all the complexes were structurally stable after binding with the ligands, with Evodiamine and Cannabinol producing the most stable ODC backbone structures, whereas β-sitosterol produced the highest backbone deviations.

**Figure 11:**
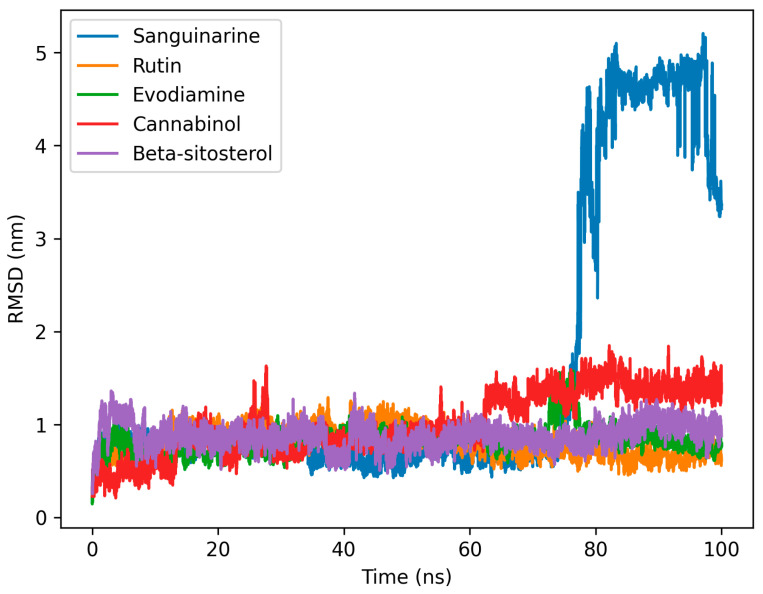
The RMSD values for the ligands Sanguinarine, Rutin, Evodiamine, Cannabinol, and β-sitosterol were examined during a 100 ns molecular dynamics simulation period. The ligands Rutin and Evodiamine showed consistently low RMSD values, suggesting higher binding stability, while the ligand Sanguinarine showed a significant increase in RMSD value, suggesting a displacement of the ligand from its binding site.

The ligand RMSD results indicated that the five complexes shown in Figure 11 exhibited distinct stability characteristics. The ligand in Rutin was the most stable, with an average RMSD of 0.803 ± 0.156 nm. Next was Evodiamine, with an average RMSD of 0.824 ± 0.147 nm, while the third stable ligand was β-sitosterol, which had an RMSD value of 0.885 ± 0.134 nm. Cannabinol had relatively high mobility, with an RMSD value of 1.011 ± 0.339 nm, while Sanguinarine had the highest deviation, averaging 1.583 ± 1.547 nm. During the last 20 ns, Rutin remained the most stable ligand, with a value of 0.680 ± 0.087 nm. Evodiamine and β-sitosterol remained moderately stable ligands, with values of 0.819 ± 0.079 nm and 0.978 ± 0.104 nm, respectively. Meanwhile, the ligand in cannabinol became more mobile at the final phase of simulation.

**Figure 12:**
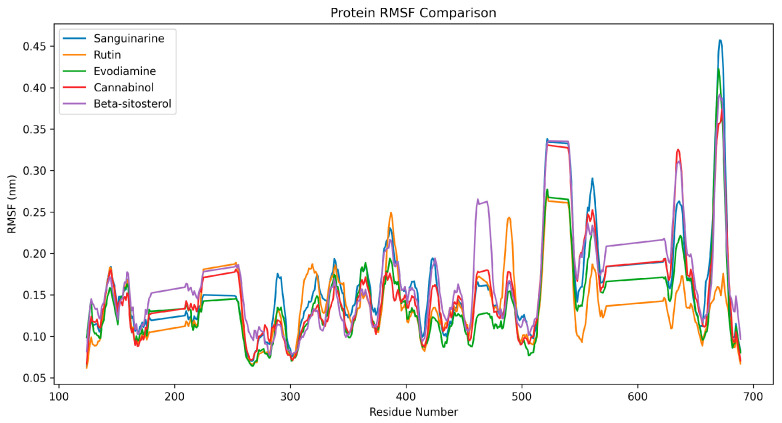
Root mean square fluctuations (RMSF) analysis of ODC in complexes with Sanguinarine, Rutin, Evodiamine, Cannabinol, and β-sitosterol was examined within a 100 ns simulation period. In general, RMSF is lower across most residues, indicating stability of the protein core, with higher peaks in flexible loops. Evodiamine and Cannabinol showed relatively lower RMSF values compared to β-sitosterol in certain regions.

Based on the RMSF analysis indicated in Figure 12, the overall protein structure remained stable across all examined structures, with fluctuation values between 0.133 and 0.158 nm per residue. In the case of ligands, the compound Rutin showed the least value in terms of mean RMSF (0.133 ± 0.058 nm), followed by Evodiamine (0.137 ± 0.070 nm), Cannabinol (0.148 ± 0.075 nm), Sanguinarine (0.156 ± 0.081 nm), and β-sitosterol (0.158 ± 0.078 nm).

**Figure 13:**
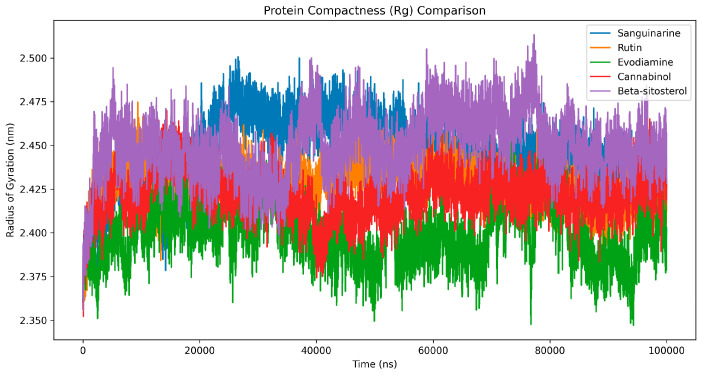
Analysis of Radius of Gyration (Rg) of ODC–Ligand Complexes. Time variation in the radius of gyration (Rg) values in the case of ODC complexes formed with Sanguinarine, Rutin, Evodiamine, Cannabinol, and β-sitosterol during a 100-nanosecond molecular dynamics simulation. ODC exhibited relatively stable Rg values, indicating that the protein remained compact. However, the value of Rg for evodiamine is relatively smaller, suggesting a more compact structure.

The Rg values (Figure 13) remained consistent throughout the simulation, indicating no change in the proteins’ global compactness. Evodiamine had the smallest Rg value (2.399 ± 0.017 nm), which means that its protein structure is the most compact among the five compounds. The structures of rutin and cannabinol were also similarly compact, with an average Rg of 2.427 ± 0.013 nm and 2.422 ± 0.013 nm, respectively.

**Figure 14:**
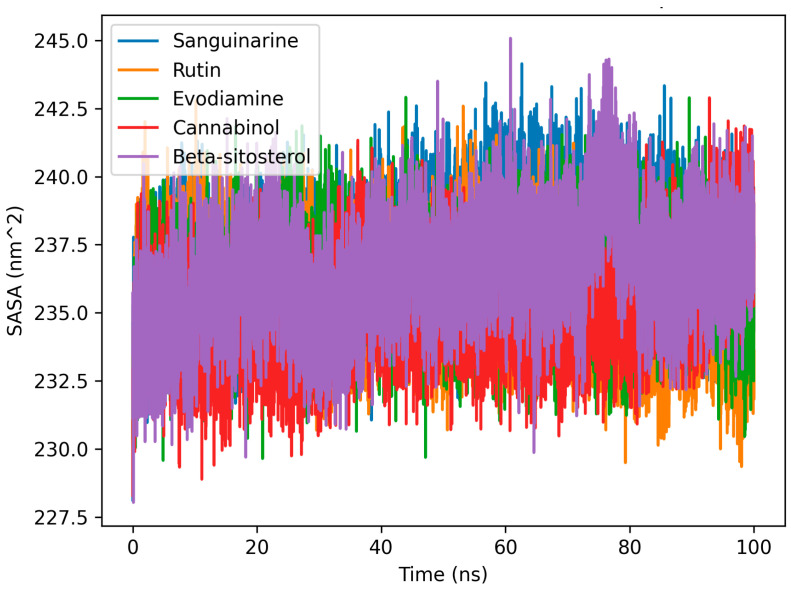
SASA Analysis of ODC in complex with Sanguinarine, Rutin, Evodiamine, Cannabinol, and β-sitosterol using 100 ns molecular dynamics simulations. All ODC-ligand complexes exhibit relatively stable SASA profiles, indicating the maintenance of protein structure and solvent exposure, with slight deviations due to conformational changes induced by the ligands.

Overall, the SASAs were stable across all complexes (Figure 14), indicating that protein folding integrity and solvent accessibility were preserved. Average SASA values were highly comparable across systems: Cannabinol (235.326 ± 1.820 nm^2^), Rutin (235.861 ± 1.747 nm^2^), Evodiamine (236.048 ± 1.705 nm^2^), β-sitosterol (236.500 ± 2.009 nm^2^), and Sanguinarine (237.209 ± 1.906 nm^2^). The narrow SASA range indicates that ligand binding did not induce major unfolding or abnormal solvent exposure.

**Figure 15:**
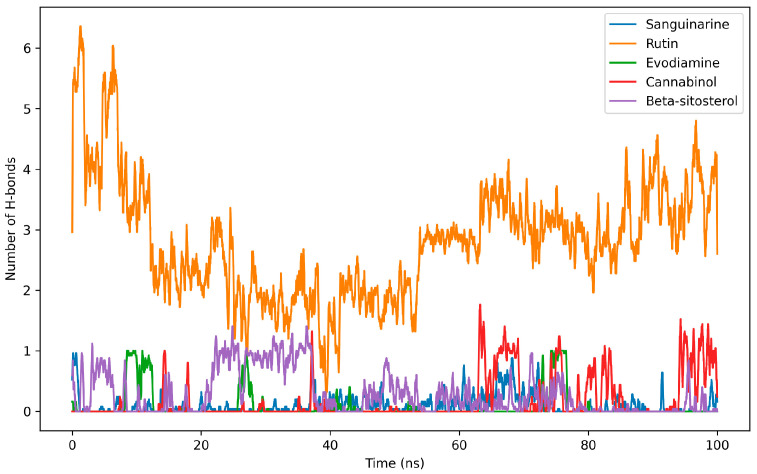
Analysis of hydrogen bonds in ODC-ligand complex structures. Changes over time in the hydrogen bonding between ornithine decarboxylase (ODC) and the ligands Sanguinarine, Rutin, Evodiamine, Cannabinol, and β-sitosterol within a 100-nanosecond molecular dynamics simulation.

Hydrogen bond analysis showed clear ligand-dependent interaction behavior (Figure 15). Rutin formed the highest number of hydrogen bonds, with an average of 2.810 ± 1.227 H-bonds across the simulation and 3.299 ± 1.039 H-bonds during the final 20 ns. This indicates persistent, stable polar interactions with the ODC-binding pocket. In contrast, Sanguinarine (0.100 ± 0.313), Evodiamine (0.080 ± 0.271), Cannabinol (0.173 ± 0.445), and β-sitosterol (0.279 ± 0.484) formed fewer hydrogen bonds, suggesting that these ligands are stabilized mainly through hydrophobic, π-based, or van der Waals interactions rather than persistent hydrogen bonding.

In conclusion, the MD simulations reveal that Rutin and Evodiamine show maximum stability with minimum ligand root mean square deviation (RMSD), RMSF, radii of gyration (Rg), and SASA values. The high hydrogen-bond occupancy observed for Rutin throughout the simulation is remarkable, whereas Evodiamine exhibits the lowest residue-level flexibility and protein radius. Although Cannabinol is structurally stable, there is an increase in ligand mobility towards the latter part of the simulation. β-Sitosterol shows satisfactory stability, with a slight increase in residue-level flexibility. Although Sanguinarine exhibits maximum docking affinity, it undergoes significant ligand displacement from its native site, suggesting that docking affinity cannot accurately predict dynamic stability.

## 4. Discussion

Ornithine decarboxylase (ODC) is a critical enzyme in the polyamine biosynthetic pathway and plays an important role in the growth, development, and maintenance of the parasite life cycle, making it a promising target for antileishmanial drug development [45,46]. While the irreversible ODC inhibitor difluoromethylornithine (DFMO) has proven effective in treating trypanosomatid infections, its inefficiency in *Leishmania* and associated shortcomings necessitate the search for alternative inhibitors with improved binding affinity and specificity [47].

There has been increased interest in natural compounds as potential antiparasitics owing to their structural diversity and ability to modulate multiple biological processes [48]. Phytochemicals isolated from *Cannabis sativa* have exhibited antimicrobial, anti-inflammatory, and antiparasitic activities, but the interactions between these phytochemicals and ODC remain largely unexplored [49,50]. In light of the above considerations, the current study proposes a combined molecular docking and molecular dynamics (MD) simulation protocol to investigate the effects of 49 phytochemicals as potential ODC inhibitors.

Sanguinarine, Rutin, Evodiamine, Cannabinol, and β-sitosterol were identified as among the most promising hits due to their binding affinities and interaction profiles. The binding energies of the complexes ranged from −7.56 kcal/mol to −8.53 kcal/mol, values consistent with recent computational research on the potential of natural product derivatives as enzyme inhibitors, implying the ability of such compounds to interact effectively with the active site of ODC. Moreover, it was noted that aromatic and polar ligands, such as Rutin and Sanguinarine, can form hydrogen bonds and π interactions, whereas hydrophobic ligands like β-sitosterol primarily engage in van der Waals and alkyl interactions. All in all, these results suggest that ODC can accommodate diverse ligands through a variety of interactions.

However, despite providing an accurate representation of binding affinity, docking cannot accurately predict ligand stability under dynamic conditions. By employing MD simulations in the study, we gained insight into ligand activity. A significant deviation exists between the affinity values obtained through docking and stability achieved in dynamic conditions, since, despite being among the compounds that bound most strongly, Sanguinarine showed considerable ligand displacement, as seen from high RMSD values. This result confirms previous observations that docking alone cannot predict ligand dynamics.

Indeed, the global structural parameters, such as the radius of gyration and solvent-accessible surface area, were relatively constant across all complexes. In other words, the global structure did not undergo any significant conformational changes due to ligand binding. Thus, we can say that the difference in inter-ligand interactions is solely due to the nature of the localized interactions. Thus, we see that the use of both docking and molecular dynamics simulations helps us understand the significance of dynamic validation in structure-based drug-prioritized compound design. While Sanguinarine had an initial binding affinity, Rutin and Evodiamine were better suited due to their greater stability and more consistent interactions in simulations. Indeed, the results of our study are consistent with those of other studies on computer-aided drug compound discovery and design [51].

Nevertheless, some limitations remain. There is an obvious need for experimental verification of the interactions and stability trends predicted by researchers. It would be important for future studies to focus on verifying the inhibitory activity of these compounds against ODC and their medicinal value in leishmaniasis. Specifically, the current methodology did not involve docking comparisons with the natural substrate, ornithine, or existing ODC inhibitors such as difluoromethylornithine (DFMO). Moreover, only one docking program was used in the current research, while molecular dynamics were performed to evaluate interaction stability. Despite these facts, it is necessary to conduct experimental studies to confirm the results from docking analysis and demonstrate the ability of natural compounds to act as effective inhibitors of ornithine decarboxylase. To sum up, this study highlights the importance of investigating cannabis compounds for their ability to inhibit ODC activity and emphasizes the crucial role of ligand stability, not just docking affinity, in determining ligand effectiveness. It provides a sound computational basis for developing new anti-leishmanial prioritized compounds.

## 5. Conclusions

In summary, the current study employs a computational approach to identify potential inhibitors of the ODC enzyme with anti-leishmaniasis activity. The selected hit compounds exhibit stable binding within the target protein’s active site. These findings should not be interpreted as supporting the direct use of cannabis or cannabis-derived products for leishmaniasis without medical supervision; rather, the identified phytochemicals represent preliminary computational prioritized compounds that require rigorous experimental, toxicological, pharmacological, and clinical validation. Prioritized compound activity, efficacy, and safety. Experimental validation is vital to prioritized compound assessment, assessing the potential of these molecules for developing effective prioritized compounds for leishmaniasis.

## Figures and Tables

**Table 1:** Compounds of *Cannabis sativa*, showcasing the best possible candidates for anti-leishmanial prioritized compounds in terms of binding affinity, ODC.

Protein	Target Ligands	Binding Affinity	PubChem CID
Ornithine Decarboxylase	Sanguinarine	−8.53 kcal/mol	5154
	Rutin	−8.15 kcal/mol	5,280,805
	Evodiamine	−7.83 kcal/mol	442,088
	Cannabinol	−7.58 kcal/mol	2543
	Beta-Sitosterol	−7.56 kcal/mol	222,284

## Data Availability

The original contributions presented in this study are included in the article/Supplementary Materials. Further inquiries can be directed to the corresponding authors.

## Supplementary Materials

The following supporting information can be downloaded at: https://www.mdpi.com/article/10.3390/biology15151272/s1, Table S1: Molecular docking results of cannabinoid compounds (n = 19) against ODC (PDB ID: 9FSC).; Table S2: Molecular docking results of terpenoid compounds (n = 10) against ODC (PDB ID: 9FSC).; Table S3: Molecular docking results of flavonoid compounds (n = 8) against ODC (PDB ID: 9FSC); Table S4: Molecular docking results of polyphenol compounds (n = 5) against ODC (PDB ID: 9FSC).; Table S5: Molecular docking results of alkaloid compounds (n = 7) against ODC (PDB ID: 9FSC); Table S6: Complete ranking of all 49 bioactive compounds by binding affinity (kcal/mol) against ODC (PDB ID: 9FSC).

## Abbreviations

The following abbreviations are used in this manuscript:

ODC	Ornithine decarboxylase
*C. sativa*	*Cannabis sativa*
CBE	Cannabielsoin
RMSD	Root Mean Square Deviation
PME	Particle Mesh Ewald
THCV	Tetrahydrocannabivarin
MD	Molecular Dynamics
ADMET	Absorption, Distribution, Metabolism, Excretion, and Toxicity
RMSF	Root mean square fluctuations
SASA	Solvent Accessible Surface Area
