# Supplementary material for: In Silico Screening of Cannabis sativa Phytochemicals as Potential Ornithine Decarboxylase Inhibitors for Anti-Leishmanial Drug-Prioritized Compound Development
Source: Biology (Basel). 2026 Aug 3;15(15):1272. doi: 10.3390/biology15151272 (PMC13465575; doi:10.3390/biology15151272)
Supplement: Supplementary file 1 [file biology-15-01272-s001.zip › biology-4219159-supplementary.pdf]

Supplementary Material

Molecular Docking Results of 49 Bioactive Compounds against Ornithine Decarboxylase (PDB ID: 9FSC)

Table S1. Molecular docking results of cannabinoid compounds (n = 19) against ODC (PDB ID: 9FSC).

No.	Ligand	Affinity (kcal/mol)	No. of Interactions	RMSD (Å)
1	Cannabinol (CBN)	-7.588	3	0.000
2	Tetrahydrocannabivarinic Acid (THCVA)	-7.455	2	0.000
3	Delta-8-THC (D8THC)	-7.280	3	0.000
4	Tetrahydrocannabiphorol (THCP)	-7.232	3	0.000
5	Cannabidivarinic Acid (CBDVA)	-7.190	2	0.000
6	Tetrahydrocannabinolic Acid (THCA)	-6.928	1	0.000
7	Delta-9-THC (THC)	-6.913	3	0.000
8	Tetrahydrocannabivarin (THCV)	-6.891	3	0.000
9	Cannabidivarin (CBDV)	-6.887	4	0.000
10	Cannabidiolic Acid (CBDA)	-6.835	2	0.000
11	Cannabicyclol (CBL)	-6.822	2	0.000
12	Cannabielsoin (CBE)	-6.807	2	0.000
13	Cannabitriol (CBT)	-6.807	4	0.000
14	Cannabichromenic Acid (CBCA)	-6.611	3	0.000
15	Cannabichromene (CBC)	-6.607	1	0.000
16	Cannabidiol (CBD)	-6.595	3	0.000
17	Cannabigerolic Acid (CBGA)	-6.586	3	0.000
18	Cannabigerol (CBG)	-6.315	3	0.000
19	Cannabidiphorol (CBDP)	-6.265	3	0.000

Binding affinity values are expressed in kcal/mol. RMSD values are reported in Ångströms (Å). All docking performed using AutoDock Vina.

Table S2. Molecular docking results of terpenoid compounds (n = 10) against ODC (PDB ID: 9FSC).

No.	Ligand	Affinity (kcal/mol)	No. of Interactions	RMSD (Å)
1	Beta-Sitosterol	-7.568	4	0.000
2	Parthenolide	-6.800	9	0.000
3	Beta-Caryophyllene	-6.489	2	0.000
4	Guaiol	-6.413	4	0.000
5	Caryophyllene Oxide	-6.002	2	0.000
6	Limonene	-5.194	2	0.000
7	Alpha-Pinene	-4.973	1	0.000
8	Beta-Ocimene	-4.869	2	0.000
9	Myrcene	-4.711	3	0.000
10	Linalool	-4.434	5	0.000

Binding affinity values are expressed in kcal/mol. RMSD values are reported in Ångströms (Å). All docking performed using AutoDock Vina.

Table S3. Molecular docking results of flavonoid compounds (n = 8) against ODC (PDB ID: 9FSC).

No.	Ligand	Affinity (kcal/mol)	No. of Interactions	RMSD (Å)
1	Rutin	-8.156	1	0.000
2	Quercetin	-7.452	1	0.000
3	Luteolin	-7.344	4	0.000
4	Myricetin	-7.344	4	0.000
5	Genistein	-7.241	2	0.000
6	Kaempferol	-7.130	1	0.000
7	Naringenin	-7.102	3	0.000
8	Apigenin	-7.024	1	0.000

Binding affinity values are expressed in kcal/mol. RMSD values are reported in Ångströms (Å). All docking performed using AutoDock Vina.

Table S4. Molecular docking results of polyphenol compounds (n = 5) against ODC (PDB ID: 9FSC).

No.	Ligand	Affinity (kcal/mol)	No. of Interactions	RMSD (Å)
1	Rosmarinic Acid	-7.154	3	0.000
2	Curcumin	-7.010	4	0.000
3	Resveratrol	-6.607	7	0.000
4	Caffeic Acid	-6.253	8	0.000
5	Gallic Acid	-5.288	7	0.000

Binding affinity values are expressed in kcal/mol. RMSD values are reported in Ångströms (Å). All docking performed using AutoDock Vina.

Table S5. Molecular docking results of alkaloid compounds (n = 7) against ODC (PDB ID: 9FSC).

No.	Ligand	Affinity (kcal/mol)	No. of Interactions	RMSD (Å)
1	Sanguinarine	-8.537	1	0.000
2	Evodiamine	-7.838	2	0.000
3	Piperine	-7.459	6	0.000
4	Colchicine	-7.120	5	0.000
5	Berberine	-6.982	5	0.000
6	Harmine	-6.383	7	0.000
7	Capsaicin	-6.367	4	0.000

Binding affinity values are expressed in kcal/mol. RMSD values are reported in Ångströms (Å). All docking performed using AutoDock Vina.

Table S6. Complete ranking of all 49 bioactive compounds by binding affinity (kcal/mol) against ODC (PDB ID: 9FSC).

No.	Class	Ligand	Affinity (kcal/mol)	No. of Interactions	RMSD
1	Alkaloid	Sanguinarine	-8.537	1	0.000
2	Flavonoid	Rutin	-8.156	1	0.000
3	Alkaloid	Evodiamine	-7.838	2	0.000
4	Cannabinoid	Cannabinol (CBN)	-7.588	3	0.000
5	Terpenoid	Beta-Sitosterol	-7.568	4	0.000
6	Alkaloid	Piperine	-7.459	6	0.000
7	Cannabinoid	Tetrahydrocannabivarinic Acid (THCVA)	-7.455	2	0.000
8	Flavonoid	Quercetin	-7.452	1	0.000
9	Flavonoid	Luteolin	-7.344	4	0.000
10	Flavonoid	Myricetin	-7.344	4	0.000
11	Cannabinoid	Delta-8-THC (D8THC)	-7.280	3	0.000
12	Flavonoid	Genistein	-7.241	2	0.000
13	Cannabinoid	Tetrahydrocannabiphorol (THCP)	-7.232	3	0.000
14	Cannabinoid	Cannabidivarinic Acid (CBDVA)	-7.190	2	0.000
15	Polyphenol	Rosmarinic Acid	-7.154	3	0.000
16	Flavonoid	Kaempferol	-7.130	1	0.000
17	Alkaloid	Colchicine	-7.120	5	0.000
18	Flavonoid	Naringenin	-7.102	3	0.000
19	Flavonoid	Apigenin	-7.024	1	0.000
20	Polyphenol	Curcumin	-7.010	4	0.000
21	Alkaloid	Berberine	-6.982	5	0.000
22	Cannabinoid	Tetrahydrocannabinolic Acid (THCA)	-6.928	1	0.000
23	Cannabinoid	Delta-9-THC (THC)	-6.913	3	0.000
24	Cannabinoid	Tetrahydrocannabivarin (THCV)	-6.891	3	0.000
25	Cannabinoid	Cannabidivarin (CBDV)	-6.887	4	0.000
26	Cannabinoid	Cannabidiolic Acid (CBDA)	-6.835	2	0.000
27	Cannabinoid	Cannabicyclol (CBL)	-6.822	2	0.000
28	Cannabinoid	Cannabielsoin (CBE)	-6.807	2	0.000
29	Cannabinoid	Cannabitriol (CBT)	-6.807	4	0.000

No.	Class	Ligand	Affinity (kcal/mol)	No. of Interactions	RMSD
30	Terpenoid	Parthenolide	-6.800	9	0.000
31	Cannabinoid	Cannabichromenic Acid (CBCA)	-6.611	3	0.000
32	Cannabinoid	Cannabichromene (CBC)	-6.607	1	0.000
33	Polyphenol	Resveratrol	-6.607	7	0.000
34	Cannabinoid	Cannabidiol (CBD)	-6.595	3	0.000
35	Cannabinoid	Cannabigerolic Acid (CBGA)	-6.586	3	0.000
36	Terpenoid	Beta-Caryophyllene	-6.489	2	0.000
37	Terpenoid	Guaiol	-6.413	4	0.000
38	Alkaloid	Harmine	-6.383	7	0.000
39	Alkaloid	Capsaicin	-6.367	4	0.000
40	Cannabinoid	Cannabigerol (CBG)	-6.315	3	0.000
41	Cannabinoid	Cannabidiphorol (CBDP)	-6.265	3	0.000
42	Polyphenol	Caffeic Acid	-6.253	8	0.000
43	Terpenoid	Caryophyllene Oxide	-6.002	2	0.000
44	Polyphenol	Gallic Acid	-5.288	7	0.000
45	Terpenoid	Limonene	-5.194	2	0.000
46	Terpenoid	Alpha-Pinene	-4.973	1	0.000
47	Terpenoid	Beta-Ocimene	-4.869	2	0.000
48	Terpenoid	Myrcene	-4.711	3	0.000
49	Terpenoid	Linalool	-4.434	5	0.000

Compounds are ranked in descending order of binding affinity. Binding affinity values are expressed in kcal/mol. RMSD values are reported in Ångströms (Å). All docking performed using AutoDock Vina.
